# Mutation of the Cell Cycle Regulator p27kip1 Drives Pseudohypoxic Pheochromocytoma Development

**DOI:** 10.3390/cancers13010126

**Published:** 2021-01-02

**Authors:** Hermine Mohr, Simone Ballke, Nicole Bechmann, Sebastian Gulde, Jaber Malekzadeh-Najafabadi, Mirko Peitzsch, Vasilis Ntziachristos, Katja Steiger, Tobias Wiedemann, Natalia S. Pellegata

**Affiliations:** 1 Institute for Diabetes and Cancer, Helmholtz Centre Munich, Ingolstaedter Landstr.1, 85764 Neuherberg, Germany; hermine.mohr@helmholtz-muenchen.de (H.M.); sebastian.gulde@helmholtz-muenchen.de (S.G.); tobias.wiedemann@helmholtz-muenchen.de (T.W.); 2 Joint Heidelberg-IDC Translational Diabetes Program, Heidelberg University Hospital, 69120 Heidelberg, Germany; 3 Institute of Pathology, School of Medicine, Technical University Munich, Trogerstr. 18, 81675 Munich, Germany; simone.ballke@tum.de (S.B.); katja.steiger@tum.de (K.S.); 4 Department of Medicine III, University Hospital Carl Gustav Carus, Technical University Dresden, Fetscherstrasse 74, 01307 Dresden, Germany; Nicole.Bechmann@uniklinikum-dresden.de; 5 Institute of Clinical Chemistry and Laboratory, University Hospital Carl Gustav Carus, Technical University Dresden, Fetscherstrasse 74, 01307 Dresden, Germany; Mirko.Peitzsch@uniklinikum-dresden.de; 6 Chair of Biological Imaging, Technical University of Munich, Ismaninger Straße 22, 81675 Munich, Germany; jaber.malekzadeh@helmholtz-muenchen.de (J.M.-N.); v.ntziachristos@helmholtz-muenchen.de (V.N.); 7 Institute for Biomedical Imaging, Helmholtz Centre Munich, Ingolstaedter Landstr.1, 85764 Neuherberg, Germany

**Keywords:** animal model, MENX, endogenous pheochromocytoma, pseudohypoxia, PPGLs, oncometabolites, mitochondrial dysfunction, 2-hydroxyglutarate, 5-hmC, cell cycle, hypermethylation, angiogenesis

## Abstract

**Simple Summary:**

Pheochromocytomas and paragangliomas (PPGLs) can be subdivided into at least three different subgroups associated with different clinical manifestations and depending on the risk to metastasize. A shortage in human tumor material, the lack of a functional human cell line and very limited animal models were major drawbacks for PPGL research and consequently for the development of patient-tailored targeted therapies. We have previously reported that the MENX rat model develops pheochromocytoma with a full penetrance at the age of 8–10 months, however, it was unclear which human group the rat tumors modeled best. In order to characterize the rat pheochromocytomas, we analyzed gene expression, the catecholamine profile, TCA-cycle metabolism, methylation, angiogenesis, histology and mitochondrial ultrastructure. In all aspects, rat MENX pheochromocytomas resemble the features of the human pseudohypoxia group, the most aggressive one and in need of effective therapeutic approaches.

**Abstract:**

Background: Pseudohypoxic tumors activate pro-oncogenic pathways typically associated with severe hypoxia even when sufficient oxygen is present, leading to highly aggressive tumors. Prime examples are pseudohypoxic pheochromocytomas and paragangliomas (p-PPGLs), neuroendendocrine tumors currently lacking effective therapy. Previous attempts to generate mouse models for p-PPGLs all failed. Here, we describe that the rat MENX line, carrying a *Cdkn1b* (p27) frameshift-mutation, spontaneously develops pseudohypoxic pheochromocytoma (p-PCC). Methods: We compared rat p-PCCs with their cognate human tumors at different levels: histology, immunohistochemistry, catecholamine profiling, electron microscopy, transcriptome and metabolome. The vessel architecture and angiogenic potential of pheochromocytomas (PCCs) was analyzed by light-sheet fluorescence microscopy ex vivo and multi-spectral optoacoustic tomography (MSOT) in vivo. Results: The analysis of tissues at various stages, from hyperplasia to advanced grades, allowed us to correlate tumor characteristics with progression. Pathological changes affecting the mitochrondrial ultrastructure where present already in hyperplasias. Rat PCCs secreted high levels of norepinephrine and dopamine. Transcriptomic and metabolomic analysis revealed changes in oxidative phosphorylation that aggravated over time, leading to an accumulation of the oncometabolite 2-hydroxyglutarate, and to hypermethylation, evident by the loss of the epigenetic mark 5-hmC. While rat PCC xenografts showed high oxygenation, induced by massive neoangiogenesis, rat primary PCC transcriptomes possessed a pseudohypoxic signature of high *Hif2a*, *Vegfa*, and low *Pnmt* expression, thereby clustering with human p-PPGL. Conclusion: Endogenous rat PCCs recapitulate key phenotypic features of human p-PPGLs. Thus, MENX rats emerge as the best available animal model of these aggressive tumors. Our study provides evidence of a link between cell cycle dysregulation and pseudohypoxia.

## 1. Introduction

Pheochromocytomas (PCCs) and paragangliomas (PGLs), together referred to as PPGLs, are neuroendocrine tumors arising from neural crest-derived chromaffin cells. PCCs develop within the adrenal medulla, while PGLs arise at many different locations within the paraganglia of the sympathetic nervous system. Hormonal imbalances occur often in PPGL patients, and cause non-specific symptoms such as hypertension, headaches, sweat and tachycardia, which make the diagnosis of these tumors difficult. PPGLs are usually benign but they all have the potential to become malignant. Surgical resection is the standard of care for confined tumors, but when they spread to other organs (up to 10–20% depending on the genetics) they become inoperable. The lack of effective treatment for metastatic PPGLs leads to high morbidity and mortality [1].

The next generation sequencing (NGS) of PPGL genomes revealed that over 30–40% of the patients carry germline mutations in at least 17 cancer susceptibility genes, thereby showing the highest rate of inherited mutations among all cancers [2,3]. Despite the variety of mutated genes, they converge in just a few common pathways. Based on transcriptional, biochemical and epigenetic profiling, PPGLs were sub-grouped in three clusters, namely: a pseudohypoxic cluster, a kinase signaling, and a Wnt-activated cluster. PPGLs with mutations in *NF1*, *HRAS*, *RET*, *MAX*, *KIF1B*, and *TMEM127* activate MAPK/ERK or PI3K/AKT/mTOR signaling pathways, and belong to the kinase signaling cluster [2]. Mutations in *CSDE1* and *MAML3*, define the Wnt-activated cluster. The pseudohypoxic cluster is characterized by mutations in succinate dehydrogenase subunits (SDHA, SDHB, SHDC, and SDHD; in short SDHx), fumarate hydratase (FH), hypoxia-inducible factor 2α (HIF2α/EPAS1), prolyl hydroxylase 1/2 (PHD1/2) or in von Hippel–Lindau tumor suppressor (VHL) [2,3]. These mutations ultimately lead to the stabilization of HIF proteins in an oxygen level-independent manner. Accumulated HIFs exert their pro-oncogenic function by inducing angiogenesis, de-differentiation, cell proliferation, anti-apoptotic processes and extracellular matrix remodeling pathways.

Pseudohypoxic PPGLs (p-PPGLs) are the most aggressive cluster; more frequently presenting with metastases at diagnosis compared to the others [4]. Given the very limited available options, finding new therapeutic approaches for the p-PPGL is a pressing clinical need, which has been hampered so far by the lack of suitable models. Indeed, the genetic manipulation of any of the pseudohypoxic driver genes in mice did not lead to PPGL development [5]. Very recently, a rat model with the heterozygous deletion of *Sdhb* was published, where, upon post-natal gamma irradiation, macroscopic PCCs developed in 3 out of 16 animals [6]. Although this is a big step forward, the low penetrance allowed only cell line models and xenografts from these rats to be used for further experiments.

MENX-affected rats carry an endogenous frameshift mutation in *Cdkn1b*, encoding a highly unstable variant of the cell cycle regulator p27kip1 (p27). Homozygous rats develop bilateral PCCs at 8–10 months of age with full penetrance, as well as abdominal PGLs (frequency approx. 10%) [7]. We have previously shown that MENX-associated PCCs loose the expression of the phenylethanolamine-N-methyltransferase (*PNMT*), a major hallmark of the pseudohypoxic cluster [8]. Here, we extensively characterized the PCCs in MENX rats by assessing their gene expression profile, vessel architecture, angiogenic potential, metabolism, methylation pattern and mitochondrial ultrastructure. We provided evidence that MENX-associated PCCs show a pseudohypoxic phenotype, making these rats the first spontaneous model of such tumors.

## 2. Results

### 2.1. Catecholamine Secretion Profile

The diagnosis of human PPGLs relies on the biochemical evidence of excessive catecholamine production achieved typically by measuring plasma or urinary catecholamine metabolites. The type of catecholamine and its relative abundance is indicative of a specific PPGL molecular cluster: tumors of the pseudohypoxic cluster usually produce norepinephrine (NE) and/or dopamine (DA) but no epinephrine (EPI) due to a lack of *PNMT* [9].

**Figure 1:**
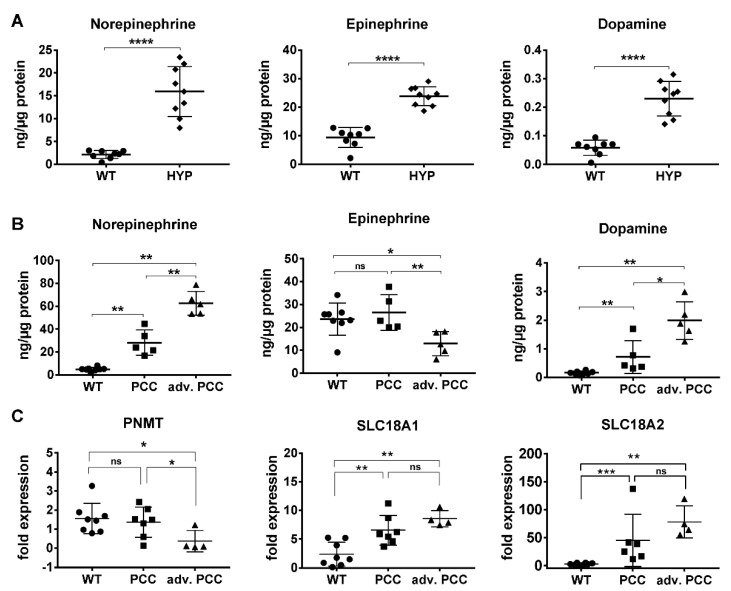
Targeted metabolomics and gene expression in rat adrenals. (**A**) Catecholamine measurements in adrenal medulla of 4–5 month-old wildtype (WT) and MENX rats with hyperplasia (HYP). Metabolites were normalized to the protein content of the gland. (**B**) Catecholamine measurements of >7-month-old WT rats, or rats with pheochromocytoma (PCC) or advanced (adv.) PCC. Metabolites were normalized as in A. (**C**) qRT-PCR-analysis of genes involved in catecholamine synthesis and transport. Depicted is the fold expression normalized against four house-keeping genes. All animals were 7–9 months old. (**A**–**C**) values + SD, *p*-values were calculated by Mann–Whitney test. (**** < 0.0001, *** < 0.001, ** < 0.01, * <0.05, ns ≥ 0.05).

We previously reported that MENX rats show a strong increase in urinary EPI, NE and the respective metanephrines compared to wildtype (WT) rats from an early age, and that the tumors lack the enzyme PNMT, which converts NE into EPI [8,10] (Figure S1A). To specifically assess the catecholamine levels in individual adrenal medullas, without the confounding factor of metabolites potentially secreted by other organs, we performed the targeted metabolomics of isolated medulla from MENX rats at 4–5 months of age (HYP) and at 7–9 months of age (PCC), and of age-matched WT rats (Figure 1).

We found a highly significant increase in EPI (8.7-fold, *p* < 0.0001), NE (7.5-fold, *p* < 0.0001) and DA (4.0-fold, *p* < 0.0001) in tissues of 4–5-month-old MENX mutant rats (HYP) vs. age-matched WT rats (Figure 1A). Given that adrenal weight increased together with age (=tumor progression), we separated the tumors into “PCCs” and “advanced PCCs”, using a cut off of ≥190 mg total gland weight for the latter. This sub-grouping allowed us to appreciate that NE and DA levels increased in both groups of neoplasms together with tumor progression (Figure 1B and Figure S1B). Indeed, NE increased +5.7-fold in PCCs vs. WT adrenal medulla (*p* < 0.002), and an average of +12.7-fold in the advanced PCC group (*p* < 0.002), while DA increased by +4.4-fold in PCCs (*p* < 0.002) and +12.2-fold in advanced PCCs (*p* < 0.002) vs. WT (Figure 1B). In contrast, EPI considerably dropped only in the advanced PCCs (−2-fold) (*p* = 0.018) (Figure 1B), which was consistent with a decrease in the expression of the *Pnmt* gene in the advanced tumor group (Figure 1C). Thus, larger tumors presented with a “NE high, DA high, EPI low” catecholamine profile.

In addition to the catecholamines, the expression of their transporters also varies among human PPGL clusters. Thus, we assessed the expression of the solute carrier family 18 member A1 (*Slc18A1*) and member A2 (*Slc18A2*), encoding VMAT1 and VMAT2, respectively, by quantitative (q) RT-PCR. Similar to human p-PPGLs, both markers were significantly upregulated in rat tumors vs. healthy WT rat adrenal (Figure 1C). In PCCs, *SLC18A1* was upregulated by a +2.8-fold (*p*-value = 0.0059) and *SLC18A2* by +17.6-fold (*p*-value = 0.0007), with a trend to further increase in advanced PCC.

### 2.2. Transcriptome Analysis 

**Figure 2:**
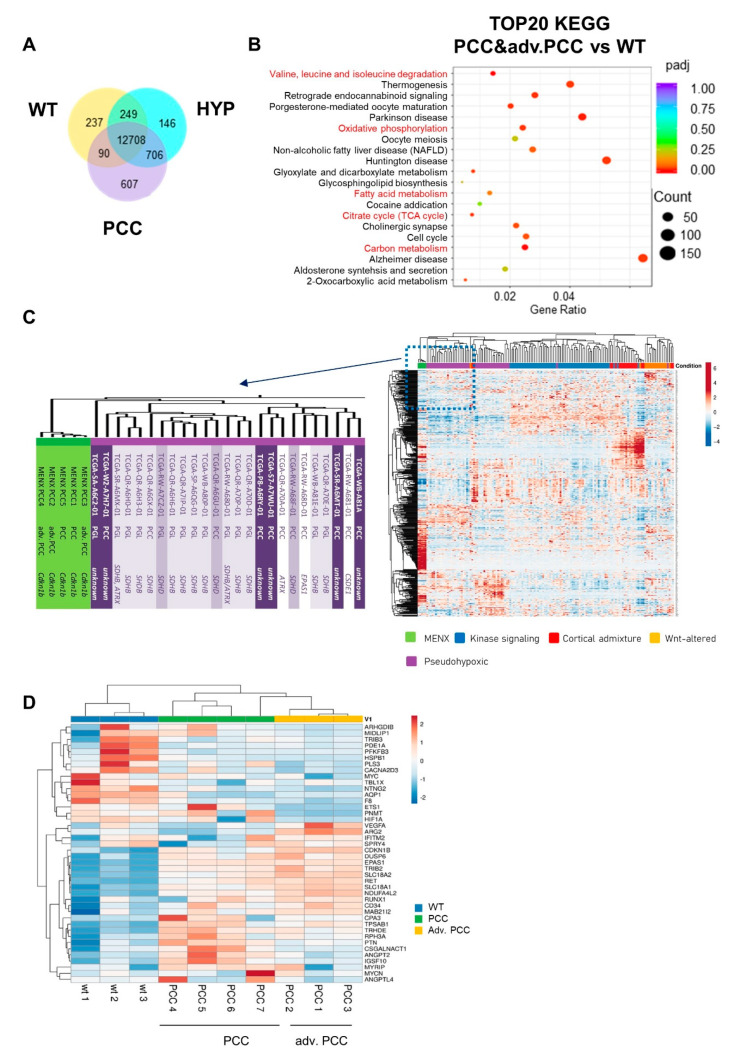
Analysis of rat transcriptome and the comparison to the human The Cancer Genome Atlas’ (TCGA) pheochromocytoma and paraganglioma (PPGL) dataset. (**A**) Venn Diagram depicting the unique and differentially expressed genes among wildtype (WT; n = 5), hyperplasia (HYP, n = 5) and pheochromocytoma (PCC + adv. PCC, n = 5) samples found by RNA-seq analysis. (**B**) Top 20 most significantly enriched KEGG pathways based on the differentially expressed genes between rat datasets (WT and PCC) are depicted in the dot graph. (**C**) Comparison of the MENX PCC transcriptome to the human TCGA PPGL dataset by the consensus clustering of the 500 most differentially expressed homologous genes between rat PCC and human PPGL. Both rows and columns are clustered using correlation distance and average linkage. The 25 patient samples closest to the rat PCCs are enlarged and their driver mutations are reported. (**D**) RT-PCR-based array analysis of selected relevant classifier genes. Shown is the consensus clustering and the heatmap of WT normal adrenal (n = 3), MENX PCC (n = 4) and advanced PCC (n = 3) of 7.5–9-month-old rats.

Human PPGLs have been originally subdivided into different clusters based on transcriptome analysis [3,11,12,13]. We performed RNA-seq of WT adrenal medulla (n = 5), HYP (n = 5) and PCCs (PCC, n = 2; advanced PCC, n = 3). Differential gene expression (Figure 2A) and the subsequent KEGG pathway analysis revealed that the most important changes among the datasets occur in metabolic processes (Figure 2B and Figure S2A,B). When comparing WT with PCC samples, among the top 20 enriched KEGG pathways were oxidative phosphorylation (OXPHOS), TCA-cycle, catabolic amino acid pathways and carbon metabolism, next to several neurodegenerative diseases (Figure 2B and Table S1). The enrichment in the metabolic pathways was to some extent already present in the HYP samples (Figure S2A), while the biggest difference between HYP and PCC was found to involve cancer-related pathways (Figure S2B).

We then compared the rat PCC RNA-seq data with the human ‘The Cancer Genome Atlas’ (TCGA) PCPG (Pheochromocytoma and Paraganglioma) dataset, containing 178 human PPGL samples by an interspecies consensus clustering of a set of 500 homologous genes (Table S2). This subset of genes was sufficient to stratify human PPGLs into the clusters previously defined by Fishbein et al. [3] (Figure S2C). The rat samples clustered together with the pseudohypoxia cluster (Figure 2C). Half of the 25 patient samples that clustered closest to the rat samples carried an SDHB mutation. The SDH complex itself was unaffected in the rats. However, MENX rat tumors mostly resemble the human TCA cycle-related subgroup of the pseudohypoxia cluster, and this is in agreement with the changes in OXPHOS, TCA cycle and carbon metabolism pathways mentioned above.

A major disadvantage of having limited availability to normal human adrenal tissue is the impossibility to determine which changes tumor cells acquire during tumorigenesis. Indeed, most studies compared human tumor samples with different genetic mutations, thus defining the subclusters mentioned above. To assess the gene expression changes between normal and tumor (medulla) tissues, we compared a subset of three WT and seven rat PCC samples of varying weight (total adrenal)/grade using a custom-made qRT-PCR array consisting of 48 genes (including four genes for normalization) selected by a literature-based rationale [3,11,14]. Among them were genes included in the 500 homologues used for the RNA-seq clustering analysis. As expected, the rat PCCs clustered according to their weight into PCCs or advanced PCCs. PCC samples 1–3, which were the largest tumors (considered advanced PCCs), showed a marked increase in relevant pseudohypoxic markers, e.g., *Epas1*, *Vegfa*, *Arg2*, *Ndufa4l2*, *Trib2*, and a decrease in *Pnmt* (Figure 2F). In the smaller PCCs (samples 4–6), these genes were also elevated vs. WT samples but to a much smaller extent. PCC7, located between the two tumor groups, likely represents an intermediate stage, as it was assigned to the early tumors by weight, but clustered closer to the advanced ones for gene expression.

### 2.3. TCA Cycle Metabolomics 

**Figure 3:**
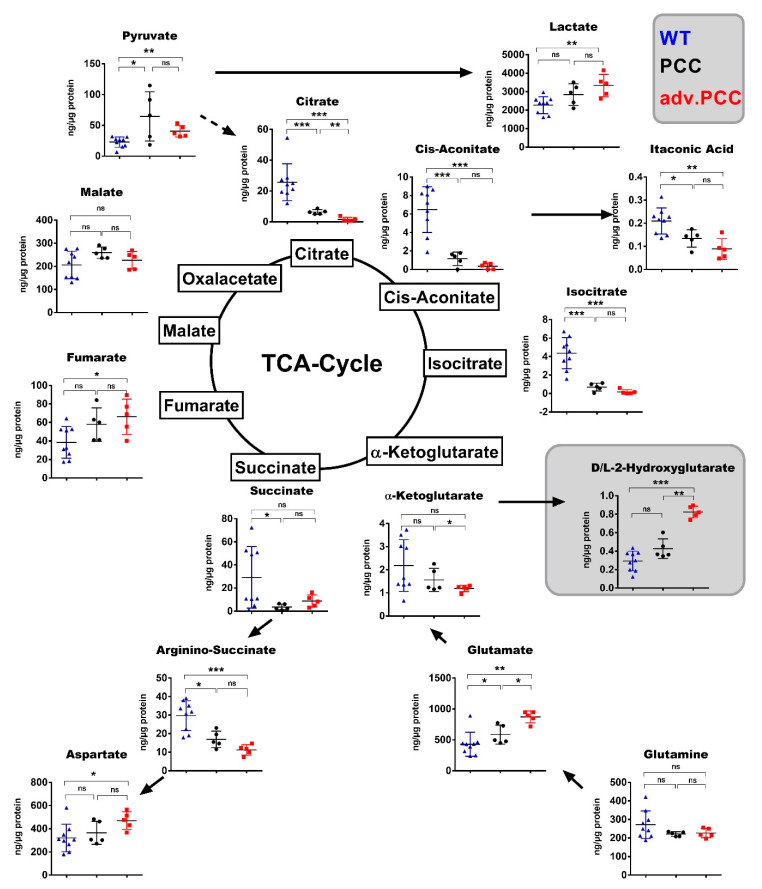
Targeted metabolomics of TCA-cycle metabolites in MENX PCCs. Metabolomic analysis of TCA-cycle metabolites in the adrenal medulla of WT (n = 9), PCC (n = 5) and adv. PCC (n = 5) at 7–9 months of age was done by LC–MS/MS. Measurements are reported as ng/g of total protein. Each symbol represents one animal: blue triangle = WT; black dot = PCC; red square = adv. PCC. Data are expressed as the mean ± SD. *p*-values were calculated by the Mann–Whitney test. (*** *p* < 0.001, ** *p* < 0.01, * *p* <0.05, ns *p* > = 0.05.

As we found various differentially expressed genes involved in metabolic processes, we performed targeted metabolomic analysis of the adrenals from 4–5-month-old and 7–9-month-old cohorts and from the respective age-matched controls (Figure 3 and Figure S3A). While succinate levels decrease by −2.0-fold (*p* = 0.0499) in the HYP (Figure S3A) and by −8.0-fold (*p* = 0.0290) in PCCs (Figure 3), in both HYP and adv. PCC lactate increased by +2.27-fold (*p* = 0.0002) and +1.45-fold (*p* = 0.0030), respectively, speaking for enhanced glycolysis. The strongest changes were seen in the levels of citrate, iso-citrate and cis-aconitate that decreased in the HYP stage and even further in adv. PCC (citrate: −16.0-fold, *p* = 0.0079; cis-aconitate: −18.5-fold, *p* = 0.0010; iso-citrate: −24.1-fold *p* = 0.0010). α-ketogluarate (α-KG) was, however, modestly reduced by −1.84-fold in adv. PCC (*p* = 0.0190). All metabolic changes are schematically summarized in Figure S3B.

The above presented data suggest increased glycolysis and decreased OXPHOS in these lesions, in line with the downregulation of TCA-cycle enzymes found in the RNA-seq analysis (Figure 2D). Some of the changes were already evident in the HYP (vs. WT adrenals) and became even more pronounced during the tumor progression (e.g., lactate, citrate, iso-citrate, cis-aconitate). In contrast, fumarate and 2-hydroxyglutarate (2-HG) levels were not altered in HYP but increased in PCCs. These metabolites play a key role in pseudohypoxic signaling with a profound impact on cancer-associated pathways [15]. Thus, during tumor progression, the metabolic profile evolved toward the typical profile of human p-PPGLs. Interestingly, the initial metabolic changes in MENX rats preceded the tumor formation.

### 2.4. Methylation 

**Figure 4:**
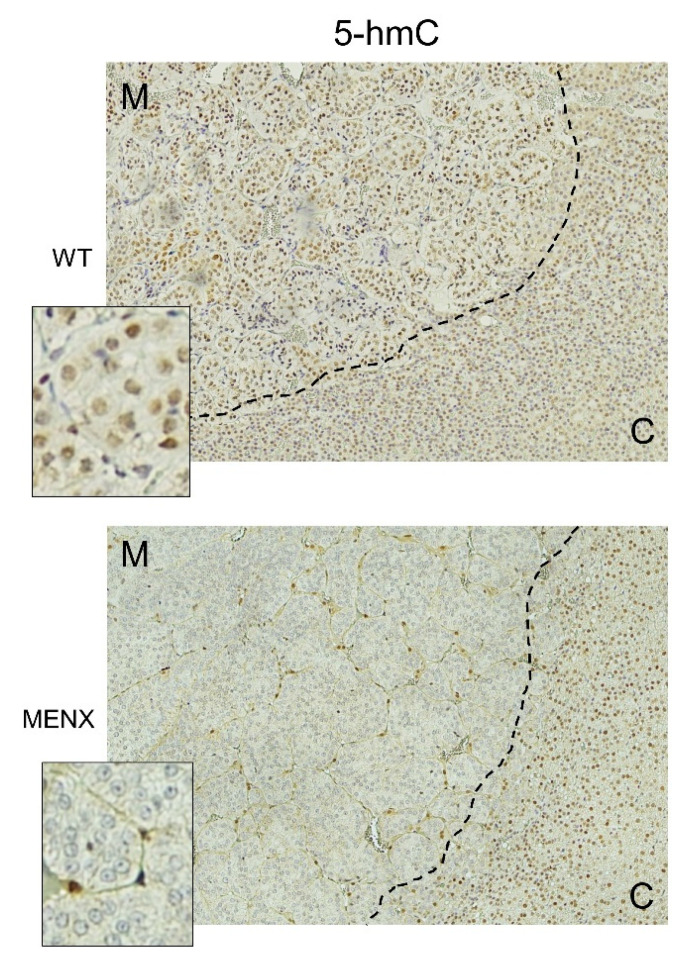
IHC of the epigenetic marker 5-hmC in MENX PCCs. IHC staining for 5-hmC of a wildtype adrenal medulla (WT) and a representative MENX adv. PCC (n = 10) at 7–9 months of age. Sustentacular cells and adjacent cortical cells remain 5-hmC positive, which is lost in the tumor cells. Magnification: 100×, inset = 300×, M = medulla, C = cortex.

In a physiological state, ten-eleven translocation (TET) enzymes hydroxylate 5-mC to 5-hmC using α-KG, oxygen and Fe2+, releasing succinate and CO2 as byproducts. This reaction was blocked by the accumulation of succinate, fumarate, or the structural α-KG-analog, 2-HG [16,17] (Figure S4A). An established marker of DNA hypermethylation was the loss of the epigenetic mark 5-hmC [16,17,18]. Given that we found a decrease in α-KG, and an increase in fumarate and 2-HG in rat PCCs, we assessed changes in 5-hmC levels by immunohistochemistry (IHC) with a specific antibody. While control adrenomedullary cells in WT rats showed a strong 5-hmC nuclear expression, rat PCC cells lacked 5-hmC positivity (Figure 4).

The loss of immunoreactivity to the anti-5-hmC antibody is specific to the tumor cells, as adjacent cortical and sustentacular cells showed a strong nuclear positivity. Chromatin hypermethylation was not a feature of adrenomedullary cells per se, but rather of tumor progression, as the cells of younger MENX rats, without pathological changes in the adrenals, were 5-hmC positive (Figure S4B). A similar hypermethylator phenotype has been previously reported for p-PPGL, and especially for SDHx-mutated tumors [19].

### 2.5. Tumor Neoangiogenesis 

**Figure 5:**
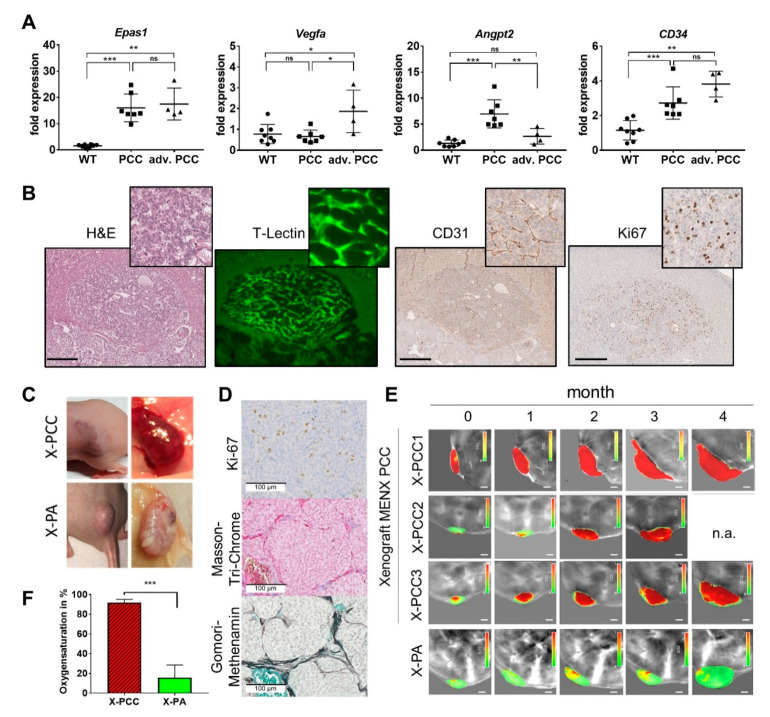
Angiogenesis-related genes and angiogenic features of rat PCCs and of engrafted rat primary PCC cells. (**A**) qRT-PCR-analysis of genes involved in angiogenesis. Depicted is the fold expression relative to a set of four house-keeping genes in WT (n = 8), PCC (n= 7) and an advanced PCC (n = 4) of 7–9-month-old rats. (**B**) Co-registration of 3D microscopy with hematoxylin–eosin (H&E), the IHC staining of a rat PCC nodule and T-Lectin labeling of intact vessels. Shown is an optical section of a 3D stack. Scale bar = 200 µm. (**C**) Xenografts of primary PCC and pituitary adenoma (PA) cells from MENX rats in immunocompromised nude mice. (**D**) IHC analysis of a representative xenografted PCC tissue bar = 100 µm. (**E**) Longitudinal multi-spectral optoacoustic tomography (MSOT) measurements of PCC (X-PCC) and PA (X-PA) xenografts assessing oxygen saturation via oxyHb/(Hb + oxyHb) ratio. The red and green colors indicate the high and low oxygen saturation, respectively. (**F**) Quantitative analysis of the oxygen saturation in tumor xenografts at the 3 months timepoint of the MSOT measurement. Depicted is mean of 3 individual tumors (**A**,**F**) +/− SD, *p*-values were calculated by the Mann–Whitney test. (**** < 0.0001, *** < 0.001, ** < 0.01, * <0.05, ns > = 0.05).

In p-PPGLs, the stabilization of the HIF2α transcription factor promotes the transcription of genes involved in angiogenesis, including Vegfa, the major pro-angiogenic factor, and Angiopoietin-2 (Angpt2), a cytokine involved in vascular remodeling [20]. In rat PCCs, concordant with a strong increase in *Epas1* expression (+10.8-fold, *p* = 0.0007), we also observed a high induction of angiogenic gene expression vs. normal adrenomedullary cells (Figure 5A). While the *CD34* (endothelial neo-angiogenesis marker) expression increased with tumor progression (PCC +1.8-fold, *p*= 0.0003; advanced PCC +3.3-fold, *p* = 0.004), *Vegfa* was elevated only in advanced PCCs (+2.4-fold, *p* = 0.0121) and *Angpt2* only in early-stage PCCs (+5.2-fold, *p* = 0.0003).

To confirm that this gene expression profile is indeed associated with angiogenesis, we visualized the tumor vasculature of rat PCCs in a vital state by 3D light-sheet fluorescence microscopy. We performed the vascular perfusion of MENX rats having tumors (n = 3) and of age-matched WT rats (n = 2) using fluorescent T-Lectin, which binds to endothelial cells. Light-sheet microscopy followed by 3D tissue rendering demonstrated that rat neoplasms have a complex neo-vascular network, where the tubes have irregular sizes and are chaotically organized instead of lining up neatly like healthy capillaries (Figure 5B and Figure S5, Video S1). The CD31 staining of endothelial cells was performed to visualize the tumor vasculature. Tumor nodules possessed high proliferation rates, as demonstrated by Ki67 staining. These rates were higher than in typical human PPGLs, and likely due to the defect in p27 (Figure 5B).

Tumor development heavily relies on the interaction between elements in the tumor microenvironment and tumor cells. To verify whether rat PCC cells are intrinsically able to promote angiogenesis due to their gene expression signature, we established the xenografts of these cells in immunocompromised mice by subcutaneous injection. We followed tumor growth longitudinally using functional multi-spectral optoacoustic (MSOT) imaging. MSOT allows to monitor the important physiological parameters of the tumors such as blood oxygen saturation (SO2) non-invasively and without contrast agent [21]. The injected rat primary PCC cells had an engraftment rate of 85%. When xenografts reached a volume of 0.5 mm^3^, tumor growth was followed by MSOT imaging until the mice met the criteria for euthanasia. Rat PCC xenografts (X-PCC) were highly vascularized tumors (Figure 5C) having histopathology and IHC similar to the tissue of origin (Figure 5D and Figure S6). In parallel, for comparative purposes, rat pituitary tumor cells xenografts (X-PA) were generated and imaged using MSOT (Figure 5C,E). To calculate the average SO2, we compared three tumors each from X-PCC and X-PA. Due to the rich blood supply, X-PCCs had 90% SO2, in contrast to 15% SO2 in the compact hypoxic X-PA (Figure 5F). In contrast to the pseudohypoxic X-PCCs, X-PAs, which possessed less angiogenic potential, evoked real hypoxia and will be described in detail elsewhere. These data demonstrated an intrinsic ability of primary rat PCC cells to generate tumors in nude mice that are highly vascularized and have high oxygen saturation.

### 2.6. Histopathology

**Figure 6:**
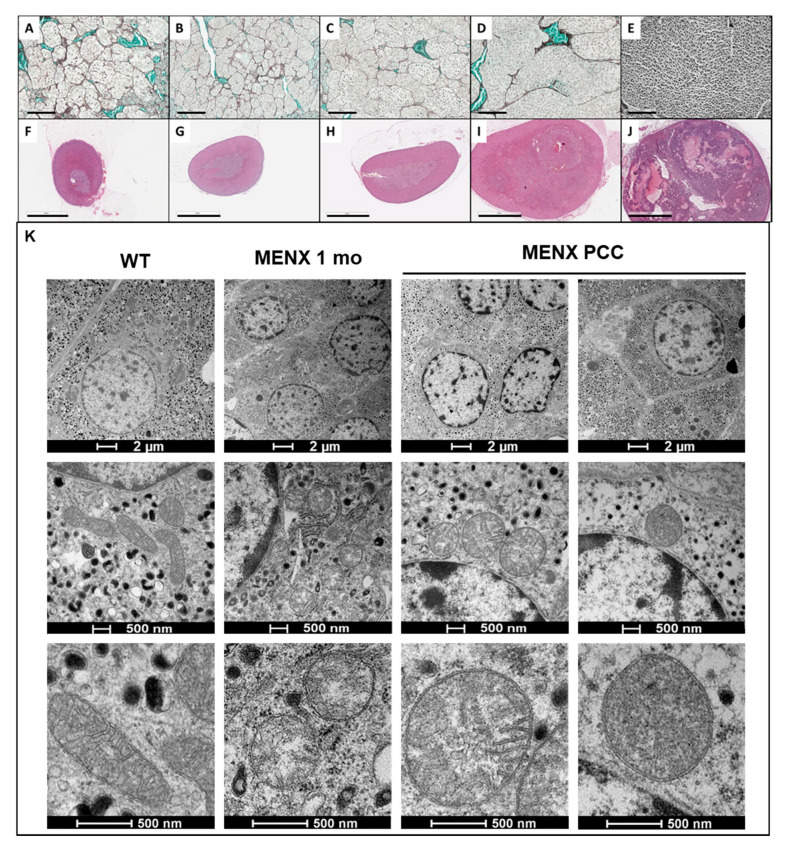
Histo- and ultrastructural morphology of MENX rat adrenals at various ages. (**A**–**E**). Reticulin network stained by Gomori–Methenamin–Silver of (**A**) WT rats, 8 months old (**B**) MENX rats, 1–2 months old (**C**) MENX rats, 4–5 months old (**D**) and (**E**) MENX rats, 7 months old. Reticulin fibers in black, erythrocytes in green. bar = 100 µm. (**F**–**J**) H&E staining of the tissues shown in panels (**A**–**E**). At >7 month of age, 100% of the adrenals were present within neoplasia, however, the manifestation varies. Extreme cases as seen in (**J**) occur in <10% of the MENX PCC cases, while (**I**) depicts a representative example of the majority of the cases at 7 mo. bar = 2 mm; (**K**) Mitochondrial abnormalities in MENX PCC. Adrenal medullary tissue of WT 1-month-old rats and PCC of 7-month-old rats were analyzed by TEM. Scale bar depicts 2 µm top, 500 nm middle and bottom panel.

In order to better understand the architecture of the rat PCC and their progression, we systematically evaluated HYP and PCC with histomorphological criteria that are applied to human PPGLs. The best known scoring systems for human PPGLs are the PASS Pheochromocytoma of the Adrenal gland Scaled Score (PASS), which includes 12 different histologic parameters covering morphology, invasion and proliferation [22] and the Grading system for Adrenal Pheochromocytoma and Paraganglioma (GAPP), considers histologic features together with Ki-67 proliferation index and catecholamine secretion profile [23]. Although their predictive value is still debated, they provide a systematic approach to characterize PPGL histopathologic features. Even though rat PCCs showed features that belong to either PASS or GAPP, none of these two scoring approaches alone were able to fully explain the phenotype of the rat tumors. Therefore, to classify MENX PCCs, we assessed the histomorphological parameters reported in Table 1, which belong to both human scoring systems. Thirty-one adrenal glands of MENX-rats were histomorphologically analyzed at different time-points and compared to those of WT rats (Figure 6A,F; Table 1).

The adrenals of the MENX rats showed a continuous progression from medullary HYP to tumors. We observed an enlarged medulla and increased nest size, visible as an expansion of the reticulin network and an increasing cellularity already at 1–2 months (Figure 6B,G). The earliest appearances of PCC in the hyperplastic context was detectable at 4–5 months (Figure 6C,H), followed by full blown neoplasia at 7–10 months (Figure 6D,E,I,J), with a loss of the reticulin network and tumors extending deeply into the cortex and replacing the entire medulla with a marked compression in the periphery. We previously reported that the Ki67 proliferation index of rat PCCs varies but is in general higher than in typical human PPGLs, likely because of the defect in p27 and cell cycle regulation in MENX rats [24]. In agreement with these studies, we observed that the Ki-67 proliferation index in PCCs ranged from 4 to 60% (Figure 5B) and was already elevated in some HYP. MENX PCCs grew either as large definite nodules, or with a more diffuse pattern and smaller nodule formation. Hemorrhage and dilated blood vessels, as a result of the chaotic neo-angiogenesis, occurred in nearly all PCCs, while the spindle-cell morphology of >10% of the tumor cells (2/23) (Figure 6E), or invasion through the adrenal capsule (3/23) occurred only in very large tumors (Figure 6J). Invasion into the surrounding tissue and undifferentiated morphology (spindle cell morphology) as well as a high proliferation rate observed in some of the neoplasia in the oldest group of animals are indicative of aggressive potential. The age of 4–5 months was seemingly the critical time point, when the switch from HYP to PCC occurred. Altogether, from a histopathological point of view, MENX-associated PCCs resemble their cognate human tumors by showing a typical Zellballen-like architecture with the formation of large irregular cell nests with increased cellularity.

### 2.7. Mitochondria Morphology

As RNAseq and metabolomics indicated changes in the adrenal medulla already at the HYP stage—thus before histopathological changes occurred—we thought to search deeper into the ultrastructural morphology of the cells. Aberrant mitochondria morphology has been reported in SDHx-mutated tumors [25], the tumor with which MENX PCCs have a high overlap based on the above presented catecholamine secretion (Figure 1), RNA-seq profile (Figure 2), methylation (Figure 4) and angiogenesis (Figure 5). We performed electron microscopy on the adrenal glands of WT (controls) and MENX rats at various ages focusing on chromaffin cells. In agreement with published studies [26], the chromaffin cells of WT rats contained mitochondria of two different morphologies: long and elongated or small ovoid, with clear-structured cristae (Figure 6K). Adrenomedullary cells of 1 month old MENX already contained mainly small ovoid mitochondria with only sparse elongated ones. Again, progression to a more severe phenotype occurred over time. Thus, in PCCs (7 months), the mitochondria were larger in size, showed balloon-like enlargements, had incomplete cristae and a blurred or empty matrix, resembling what has been described for SHDx-mutated human PPGLs [17].

## 3. Discussion

We here provide extensive evidence that PCCs MENX rats recapitulate the most important features of human p-PPGLs. These rats represent the only currently available animal model where such tumors develop in their physiological microenvironment with complete penetrance.

MENX rats carry a spontaneous mutation in *Cdkn1b* encoding p27 [7]. In humans, germline p27 mutations cause the MEN4 syndrome [27], where the involvement of adrenal glands is rare [28]. In sporadic PCCs, no loss-of-heterozygosity LOH at the *CDKN1B* locus nor gene mutations were identified, but we found p27 to be downregulated or lost in 56% of sporadic PCCs [29]. By means of MENX genetics, it was not immediately obvious which human subcluster the rat tumors might represent. Former studies by our group suggested that MENX-associated PCCs lack PNMT, a feature of noradrenergic tumors, but no in-depth analysis had been done [8]. Thus, we set out to systematically assess the histomorphological, molecular, and biochemical features used to classify human PPGLs. Using this approach we not only found the high overlap with the human p-PPGL cluster but also obtained new insights into the events of tumorigenesis.

RNA-seq data analysis unequivocally clustered MENX PCCs together with human p-PPGL. The expression of many classifier genes of the pseudohypoxic cluster (i.e., *Epas1*, *Slc18A1/2*, *Ndufa4l2*, *Vegfa*) increased with the size of the rat tumors suggesting that they play an important role in tumor progression, but not necessarily in tumor initiation. Accordingly, catecholamine metabolomics of individual rat adrenal glands with advanced PCCs identified the typical pseudohypoxic profile: NE high, DA high, EPI low, which are very important classification criteria for human p-PPGL. Rat PCCs had lower levels of the TCA cycle metabolites citrate, iso-citrate and cis-aconitate, regardless of their stage of progression, as previously shown for human p-PPLGs [30,31]. Already at the HYP stage, rat lesions showed changes in OXPHOS, carbon metabolism as well as in several anabolic amino acid pathways by RNA-seq and metabolomics, implying a dysfunction in energy metabolism. In contrast, fumarate and the onco-metabolite 2-HG were elevated only in late-stage tumors, suggesting that, in MENX rats, they are not the initiators of tumorigenesis but rather represent the secondary events associated with tumor progression. Interestingly, by ultrastructural analysis, the adrenomedullary cells of mutant rats showed morphologically altered mitochondria already at the age of 1 month, before the onset of histo-pathological changes. Mitochondrial abnormalities in mutant rats worsened with age, and in the tumor cells of 7-month-old MENX rats, mitochondria showed balloon-like enlargements and incomplete cristae. The shape and organization of the cristae affects the functionality of the electron transport chain and thus OXPHOS performance [32]. This is indeed what we observe in MENX PCCs, where both mitochondrial morphology and functionality are affected. At present, we cannot determine which of these two events occurred first and triggered the other. Additional analyses, e.g., whole genome sequencing or metabolic flux analyses, are required to determine whether somatic mutations and/or deteriorating mitochondrial function lead to the accumulation of oncometabolites (fumarate, 2-HG) in rat PCCs.

SDHx- and FH-mutated human p-PPGL lose the epigenetic marker 5-hmC and this associates with DNA hypermethylation [19]. These epigenetic alterations interfere with transcriptional regulation by modulating chromatin accessibility. TET enzymes regulate the conversion from 5-mC to 5-hmC, and their activity is inhibited by increased levels of fumarate, succinate or 2-HG. This links the mitochondrial defects of p-PPGLs bearing mutations in the TCA-cycle with epigenetic modification, and ultimately with gene regulation. Interestingly, like in human SDHx PPGLs, 5-hmC is lost in rat tumor cells but not in adjacent normal medullary cells. Thus, the increase in fumarate and 2-HG together with the decrease in α-KG in MENX PCCs likely block the functionality of TET enzymes, resulting in the loss of the 5-hmC mark. It remains to be determined whether the loss of 5-hmC is also associated with aggressive behavior and a worse patient outcome in human PPGL, as found in other hypoxic and pseudohypoxic cancers such as clear cell renal cell carcinoma (ccRCC) [16] and melanoma [33]. In this respect, it is worth to note that in the imCC cell line [19,34,35], a spontaneously immortalized mouse chromaffin cell line with full Sdhb knockout, the hypermethylation phenotype could be induced by TET silencing [34,35]. In combination with HIF2α activation, this resulted in an epithelial-to-mesenchymal transition mimicking metastatic behavior in human p-PPGL [34,35].

We observed that rat PCCs are mainly negative for the expression of the enzyme PNMT, as previously reported [7]. The rodent adrenal medulla, in contrast to the human medulla, contains two subpopulations consisting of PNMT-positive and PNMT-negative cells arranged in small clusters. At this stage, it is not clear whether the MENX neoplasms originate from PNMT-negative chromaffin cells, or whether during tumor progression *Pnmt* expression is lost due to promoter methylation. Such a mechanism has been reported in human P-PPGLs: the *PNMT* promoter was found to be the target of TET-mediated hypermethylation and resulting in decreased gene expression [19]. Interestingly, it has been shown that rats treated with vitamin D3 developed adrenomedullary hyperplasia and PCCs [36], which were mainly PNMT-negative, thereby suggesting that norepinephrine cells are more sensitive to mitotic stimuli. Currently, no conclusive statement can be made about what causes the lack of *Pnmt* expression in the rat tumors. A combination of both mechanisms (cell of origin, expression downregulation) might be possible.

Pathomorphologically, MENX PCCs share many characteristics with human PCCs, as defined by the PASS or GAPP scoring systemS. In patients, no histo-morphological criteria can clearly distinguish benign from malignant tumors with 100% confidence. However, in human PPGLs, there are features suggestive of aggressive tumor behavior, including local invasion and undifferentiated cell morphology together with other morphological markers. In advanced rat PCCs, we also found the invasion of the periadrenal adipose tissue and cell spindling with increased tumor cellularity. However, no distant lymph node metastases of PCCs have been identified in MENX rats so far. A reason for this might be that, due to their deteriorating health, these animals have to be sacrificed before neoplasias spread to other organs.

In agreement with the hypothesis that unstructured tumor vessels can promote the progression to invasive and metastatic tumors [37], chaotic vessel architecture has been described for human malignant PPGLs [20]. To verify whether this also applies to MENX PCCs, we visualized the architecture of the blood vessels in intact adrenal glands in 3D using T-Lectin labelling and light-sheet microscopy [38]. When compared with normal adrenal medulla, rat tumor nodules showed a rich, highly tortuous, and disorganized vessel network. In agreement with their high degree of vascularization, rat PCCs (early and late) express high levels of *Epas1* and *CD34* vs. normal adrenal medulla. In addition, early tumors display elevated *Angpt2* expression, whereas *Vegfa* is increased only in late-stage PCCs. The ability of rat PCC cells to promote angiogenesis is cell-autonomous, given that when engrafted into nude mice they generate tumors that are highly vascularized and have high oxygen saturation, as demonstrated by MSOT. This is consistent with the expression of the pro-angiogenic factors mentioned above (Hif2α, Angpt2 and Vegfa). Thus, in addition to the histological features of aggressiveness, advanced rat PCCs display a vessel architecture reminiscent of malignant human PPGLs, and represent a useful platform to evaluate the efficacy of anti-angiogenic drugs against these tumors (see below).

Our findings that the mutation of p27 predisposes to PCCs having an alteration in the energy metabolism can be interpreted in the context of a signaling mechanism that connects cell cycle checkpoints to mitochondrial function [39]. The dysregulation of mitochondrial function in human p-PPGLs is associated with severe oxidative stress due to increased reactive oxygen species (ROS) production [40]. It is noteworthy that the oxidative stress response mediated by Foxo3a induces p27 transcription, which ultimately leads to cell-cycle arrest [41], thereby preventing cell division in the presence of unbalanced ROS levels [42]. Thus, to explain the metabolic changes found in rat PCCs, one could postulate that p27 regulates mitochondrial metabolism via an as of yet unknown feed-back loop, which is defective in MENX PCC cells having no functional p27 protein [26]. Interestingly, Powers et al. found that dissected normal rat adrenal medulla clusters together with human p-PPGL transcriptomes [6]. Therefore, the activation of the pseudohypoxic pathways in MENX tumors could be explained by an intrinsic ability of chromaffin cells to sense oxygen. In this case, the lack of the functional p27 would not need to induce novel oxygen-responsive pathways in these cells but rather stimulate the already existing ones. Further studies are required to address these points.

After various unsuccessful attempts at generating engineered mouse models of pseudohypoxic tumors, recently, a rat line with a heterozygous Sdhb deletion was established by TALEN-genetic engineering [6]. As in mice, the homozyogous deletion of Sdhb in rats led to embryonic lethality, but unlike the mice, Sdhb+/− rats developed PCCs, albeit at low frequency [6,43]. After neonatal irradiation, the frequency of macroscopic tumors in Sdhb+/− rats increased to 20%, indicating that additional events were necessary for tumorigenesis. Unfortunately, the low incidence/lack of tumors in nearly all previous mouse/rat models of p-PPGLs hampers their usage for preclinical therapy studies.

Several novel therapeutic approaches for p-PPGLs await preclinical testing, and, thanks to their unique characteristics, MENX rats represent a unique platform for such studies. Indeed, although these rats are not a model of SDHx driver mutations, they are highly valuable given the extensive phenotypic overlap with pseudoxypoxic tumors. Among the drugs potentially effective against p-PPGLs are Hif2α inhibitors, which to date have been tested against ccRCC [44], and therapeutics targeting DNA methylation such as DNA methyltransferase inhibitors or histone deacetylase inhibitors. MENX rats represent the ideal model to evaluate the efficacy of these compounds as their tumors express high levels of *EPAS1* and show loss of 5-hmC.

In conclusion, despite having a different driver gene mutation, MENX-associated PCCs recapitulate all key characteristics of p-PPGLs. Moreover, MENX rats show a time-dependent tumor progression going from HYP to early, and finally advanced lesions. The possibility to study PCCs at different stages of tumorigenesis is not applicable to human patients and may give important clues as to the early changes promoting adrenomedullary carcinogenesis, as well as provide biomarkers for early diagnosis or detection. Importantly, advanced rat PCCs have features that, in their cognate human tumors, are indicative of malignant potential. Understanding the molecular mechanisms leading to these tumors may create novel therapeutic opportunities for aggressive PPGLs but also for other pseudohypoxic cancers. The MENX line, together with complementary in vitro models, is expected to further increase our knowledge of pseudohypoxic tumorigenesis and should help filling the gap that was created by the limitations/lack of the currently available models of p-PPGLs.

## 4. Materials and Methods 

### 4.1. Animals 

MENX rats originate from a spontaneous mutation in the Cdkn1b gene encoding for p27Kip1 in a colony of Sprague Dawley rats, leading to the development of multiple endocrine tumors, including bilateral pheochromocytoma, extra-adrenal paraganglioma, pituitary neuroendocrine tumor (piNET), thyroid C-cell hyperplasia, parathyroid hyperplasia, and pancreatic islet cells hyperplasia [7]. Only primary PCC and piNETs were used for this study. All experimental procedures were conducted in accordance with committed guidelines as approved by the local government (GV-Solas; Felasa; TierschG) and general husbandry rules of the Helmholtz Centre Munich. In vivo studies were approved by the government of Upper Bavaria, Germany (Az. 55.2.1.54-2532-111-2015, ROB-55.2-2532.Vet_02-18-102).

### 4.2. Preparation of Tissue

If not otherwise stated, adrenal glands were dissected, perirenal adipose tissue was carefully removed, the total adrenal was first weighted and then the cortex was removed by macrodissection. Isolated medulla was snap-frozen in liquid nitrogen until further use. A cut off of >190 mg total adrenal was applied to divide the tumors into PCC or advanced PCC based on the results of the catecholamine analysis.

### 4.3. Mouse Xenografts

Immunocompromised mice were injected subcutaneously 1 × 10^6^ MENX primary PCC cells (n = 6) or with 1 × 10^6^ MENX primary pituitary tumor cells (n = 6). Tumor monitoring in rats was conducted longitudinally and non-invasively using multispectral optoacoustic tomography (MSOT). MSOT measurements were performed monthly after the tumor size reached a volume of 0.5 mm^3^. Mice were sacrificed when the tumor volume reached the approved criteria, or at first sign of discomfort.

### 4.4. Multispectral Optoacoustic Tomography (MSOT) 

Mouse xenografts were scanned with an MSOT scanner (MSOT256-TF; iThera Medical, Germany) over 21 wavelengths in near-infrared spectral region (700–900 nm.) [45]. The measured optoacoustic signal was reconstructed using a model-based algorithm to produce images at different wavelengths [45,46,47]. A linear un-mixing method was used to extract oxy- and deoxy-hemoglobin distribution from the reconstructed data. Blood oxygen saturation SO2 was computed by using extracted oxy- and deoxy-hemoglobin coefficients as previously reported [47]. To monitor the variations of oxy-and deoxy-hemoglobin, seven acquired cross-sectional images roughly located at the center of the tumor were chosen and the mean and standard deviation of each variable was calculated at different time points separately.

### 4.5. Tissue Embedding and Histology

Formalin-fixed and paraffin embedded adrenal glands were cut in 2 µm-thin sections with a rotary microtome (HM355S, ThermoFisher Scientific, Waltham, MA, USA) and subjected to histological and immunohistochemical analysis. Hematoxylin–eosin (H&E) staining was performed on deparaffinized sections with Eosin and Mayer’s Haemalaun according to a standard protocol. Morphological evaluation was performed on H&E-slides referring to the grading of Kimura et al. 2005 (GAPP) and Thompson 2002 (PASS) for histopathologic features [22,23]. Features used for evaluation are summarized in (Table S3). Reticulin-staining was performed with the “Methenamine silver plating kit according to Gomori” (Merck), according to the manufacturer’s instructions.

### 4.6. Light-Sheed Fluorescent Microscopy

Rats were anesthetized via isoflurane and injected with 6 nmol/250 gr BW (i.v.) TLectinSense 680 (Perkin Elmer; NEV10060). The rats woke up and were housed in their home cages for the next 6 h. After this incubation time, the rats were killed and immediately perfused with 0.9% cold saline. After finishing the perfusion, the organs of interest were cut out and fixed in PaxGene^®^ Fixation (PreAnalytiX; 765312) reagent for 24 h at room temperature covered in aluminum foil to prevent light exposure. Subsequently, the tissues were transferred into PaxGene^®^ Stabilizer solution and stored at 4 degrees covered with aluminum foil.

### 4.7. Clearing

Tissue clearing was done using the 3DISCO protocol [10]. Briefly, samples were dehydrated in ascending concentrations (50, 70, 80 and 100%) of Tetrahydrofuran (THF, Roth) adding molecular sieves (Sigma) at the last step. The incubation of THF 100% was done for 3 × 12 h. Samples were then immersed in Dibenzylether (DBE, Merck) until clearing was completed, but at least for 3 days. Samples were then analyzed using a light sheet fluorescence microscope (UltraMicroscope II, LaVision BioTec).

### 4.8. Histology after LSFM for Co-Localization 

Cleared tumor samples were rehydrated by immersing them in descending concentrations of ethanol solutions (100% for 24 h, 80% for 48 h, and 70% for 24 h). After rehydration, the tissue samples were again dehydrated using an automated system (Leica ASP300S; Leica Biosystems) and subsequently embedded in paraffin. Serial 2 µm-thin sections prepared with a rotary microtome (HM355S; ThermoFisher Scientific) were subjected to histological and immunohistochemical analysis. Serial sections were done in 50 µm steps. Hematoxylin and eosin (H&E) staining was performed on deparaffinized sections with Eosin and Mayer’s Haemalaun (Morphisto) according to a standard protocol.

### 4.9. Immunohistochemistry

Immunohistochemistry (IHC) was performed with an automated immunostainer (Ventana Medical Systems) or Bond RXm system (Leica) according to the manufacturer’s protocols with minor modifications. Immunostaining was performed with the antibodies indicated in Table S4.

### 4.10. Metabolomics

Tissue lysis for catecholamine and citrate cycle metabolites as well as amino acids measurements.

Snap-frozen macrodissected medullary chromaffin tissue from age-matched (4–5 months or 7–9 month) male mutant or WT MENX rats were lysed by adding 100 µL cell disruption puffer (Ambion PARISTM Kit, Thermo Fisher), 15 min of incubation at 4 °C and homogenization using a tissue grinder (NIPPON Genetics Europe, Düren, Germany) followed by another 15 min incubation on ice. After centrifugation, the obtained supernatants were diluted in either 0.4 M perchloric acid containing 0.5 mM EDTA in Milli-Q water (catecholamines measurements), methanol (citrate cycle metabolites and amino acids measurements) or water (protein determination). For normalization purposes, protein concentration from lysates was measured using Bradford Assay (Roti^®^-Quant, Roth, Karlsruhe, Germany). Data are provided in Table S5.

### 4.11. Catecholamines

Diluted lysates obtained as specified above were analyzed by high-pressure liquid chromatography (HPLC) with electrochemical detection as described previously [48]. TCA cycle metabolites and amino acids and methanolic lysates were quantified by high-pressure liquid chromatography tandem mass spectrometry (LC–MS/MS) as described previously [49].

### 4.12. Quantitative Real-Time PCR (qRT-PCR) Analysis

RNA from macrodissected medullary chromaffin tissue of MENX WT and mutants (age, as indicated in the respective experiment) was extracted by the Maxwell extraction system using the simplyRNA kit (Promega). Real-time PCR gene expression was conducted using Quantifast-One-Step RT-PCR Plus Kit (Qiagen) with customized RT-qPCR arrays or single gene-expression assays (Thermofisher) (Table S6) on a ViiA 7 Real Time PCR system (Thermofisher). A set of four genes, namely Vdac3, Srsf3, Itm2b, Mtch1, that were identified by Flynn et al. [11] to be suitable house-keeping genes for pheochromocytoma studies have been used for normalization and the 2^−ΔΔ*C*t^ method has been applied to calculate differential gene expression.

### 4.13. RNA-Seq Analysis 

RNA for RNA-seq analysis was obtained as described above. Each 5 animals of 4–5-month mutant (=HYP), 7–9.5-month mutant (=PCC), 7–9.5 month WT were used for analysis. The PCCs were subdivided in 2 PCCs and 3 advanced PCCs. Adrenal medullary tissue was macrodissected and cortex removed. RNA-seq and bioinformatics analysis was performed at Novogene (Cambridge, UK). Briefly, 1 μg total RNA, with RIN scores > 6.5 were was used for the generation of sequencing libraries using NEBNext^®^ UltraTM RNALibrary Prep Kit for Illumina^®^ (NEB, Ipswich, MA, USA) following the manufacturer’s recommendations. Fragments of 150~200 bp length were purified with the AMPure XP system (Beckman Coulter, Beverly, MA, USA) and sequenced on the Ilumina platform in paired-end reads. Clean reads were mapped to the reference genome Rn06 Rattus norvegicus (downloaded from NCBI/UCSC/Ensembl) using HISAT2 software. Differential expression analysis was performed with DESeq2 package and the KEGG database were used for enrichment pathway analysis. Datasets for this study were deposited into the Gene Expression Omnibus (GEO) database (http://www.ncbi.nlm.nih.gov/geo/) under accession identification number (GSE162970).

### 4.14. Consensus Clustering

The data of the TCGA PPGL samples from the harmonized PCPG dataset were downloaded via the Bioconductor package TCGAbiolinks [50]. Raw read counts of rat and human samples were TPM normalized [51]. The 3000 most differential genes were searched for rat homologous using the biomaRt R package, retrieving 1699 homologs as previously published [6]. Genes with no reads were removed from the analysis. After combining human and rat normalized reads, the 500 most-variable genes by median absolute deviation were taken for consensus cluster analysis, using the ClustVis online tool [52]. A list of the genes is provided in Table S2. The appropriateness of the subset was tested by clustering human samples only first. Human TCGA cases were limited to those, which were assigned to a certain human PPGL cluster as described in Fishbein et al., 2017 [3]. Parameters applied to the clustering were as follows: original values were ln(x + 1)-transformed. Rows were centered; Pareto scaling was applied to rows. Both rows and columns were clustered using the correlation distance and average linkage. All R analyses were performed with the R (version 3.6.0) (https://www.r-project.org/).

### 4.15. Transmission Electron Microscopy (EM)

Fresh macrodissected tumor tissue was cut into 3 mm pieces and fixed with freshly prepared 2.5% (*v*/*v*) glutaraldehyde, 2% paraformaldehyde in 0.1 M sodium cacodylate, pH 7.4 for 24 h at 4 °C. Tissues were transferred to 0.1 M sodium cacodylate pH 7.4 and stored at 4 °C until further processing. Tissues were prepared as previously described [53] and imaged on a Tecnai G2 (FEI) transmission electron microscope operating at 100 kV at the EM Facility of the University of Padua. Images were captured using a Veleta (Olympus Imaging System) digital camera.

### 4.16. Statistics

qRT-PCR results and metabolomics data are presented as scattered dot plots with individual animals as points, with a mean and SD. Mann–Whitney test was performed with GraphPad Prism (GraphPad Software Inc., Witzenhausen, Germany). Significance is depicted as **** < 0.001, *** < 0.001, ** < 0.01, * < 0.05.

## 5. Conclusions

Pheochromocytoma and paraganglioma of the pseudohypoxic subtype (p-PPGLs) have the highest propensity to metastasize, and associate to poor prognosis. No approved targeted therapy currently exists for p-PPGLs. Progress toward novel therapies for p-PPGLs has been slow, owing mostly to the lack of reliable animal models mimicking the human disease. We found that PCCs in MENX rats recapitulate key features of human p-PPGLs. In addition, they allow to study the progression to aggressive tumors, which might provide early biomarkers of disease and offer novel insights in tumor biology. These aspects, combined with complete tumor penetrance, establish MENX rats as ideal preclinical model to identify and test novel treatments against p-PPGLs, potentially leading to the improved clinical management of patients with p-PPGLs or other aggressive pseudohypoxic tumors. This model can also shed light on the cross-talk between cell cycle regulation and the control of cell metabolism in tumorigenesis.

## Figures and Tables

**Table 1:** Overview of the histomorphological characteristics of MENX PCC.

	MENX (Total = 31)		
**Diagnosis**	1–2 mo (n = 4)	4–5 mo (n = 4)	>7 mo (n = 23)
Hyperplasia	4/4	3/4	1/23
Early PCC		1/4	3/23
PCC			19/23
**Major findings**			
Architecture:			
regular, nests can get wider, focal loss of reticulin network	4/4	3/4	23/23
enlarged irregular nests, diffuse, small nodule formation and loss of reticulin network		1/4	13/23
enlarged irregular nests with distinct large nodule formation and loss of reticulin network			9/23
Pleomorphism			
grade 1		2/4	22/23
grade 2			1/23
Increased cellularity	4/4	4/4	23/23
Pyknoses	2/4	2/4	21/23
Mitosis, atypical mitosis		2/4	14/23
Small comedonecrosis and/or fatty degeneration			12/23
Spindle cell architecture (>10% of tumor volume)			2/23
Invasion through the capsule into periadrenal adipose tissue			3/23

## Data Availability

The data presented in this study are available in the article or supplementary files. RNAseq data has been deposited as stated above.

## Supplementary Materials

The following are available online at https://www.mdpi.com/2072-6694/13/1/126/s1: Figure S1: PNMT expression and catecholamine profile of MENX PCCs; Figure S2: Analysis of rat transcriptome and Human TCGA PPGL clustering with the 500 gene list, Figure S3: Krebs cycle changes in hyperplastic MENX PCCs, Figure S4: TET enzymes activity and 5-hmC expression in MENX rat adrenals; Figure S5: Structure of the rat PCC vasculature. Figure S6: Comparison of the morphology of primary MENX PCCs and tumors derived from xenografted MENX PCC cells. Table S1: List of the KEGG pathway analysis in WT vs. PCC as xlsx, Table S2: List of 500 genes used for clustering as .xlsx, Table S3: Features used to access histomorphology, Table S4: Antibodies used in the study, Table S5: Metabolomics Table as .xlsx, Table S6: List of Taqman Assays as .xlsx, Video S1: 3D reconstruction of cleared-T-Lectin stained MENX adrenal with PCC. Additional datasets analyzed during the current study are available from the corresponding author upon reasonable request.

## Abbreviations

2-HG	2-hydroxyglutarate
HYP	hyperplasia
IHC	immunohistochemistry
EPI	epinephrine
LSFM	light sheet fluorescence microscopy
MSOT	multispectral optoacoustic tomography
NE	norepinephrine
OXPHOS	oxidative phosphorylation
PCC	pheochromocytoma
PGL	paraganglioma
PPGL	pheochromocyomat and paraganglioma
p-PPGL	pseudohypoxic pheochromocytoma and paraganglioma
WT	wildtype
