# Supplementary material for: Mutation of the Cell Cycle Regulator p27kip1 Drives Pseudohypoxic Pheochromocytoma Development
Source: Cancers (Basel). 2021 Jan 2;13(1):126. doi: 10.3390/cancers13010126 (PMC7794757; doi:10.3390/cancers13010126)
Supplement: Supplementary file 1 [file cancers-13-00126-s001.zip › Supplements/cancers-1015203 - supple-check.docx]

Supplementary Materials: Mutation of the Cell Cycle Regulator p27kip1 Drives Pseudohypoxic Pheochromocytoma Development

Hermine Mohr, Simone Ballke, Nicole Bechmann, Sebastian Gulde, Jaber Malekzadeh-Najafabadi,
Mirko Peitzsch, Vasilis Ntziachristos, Katja Steiger, Tobias Wiedemann and Natalia S. Pellegata


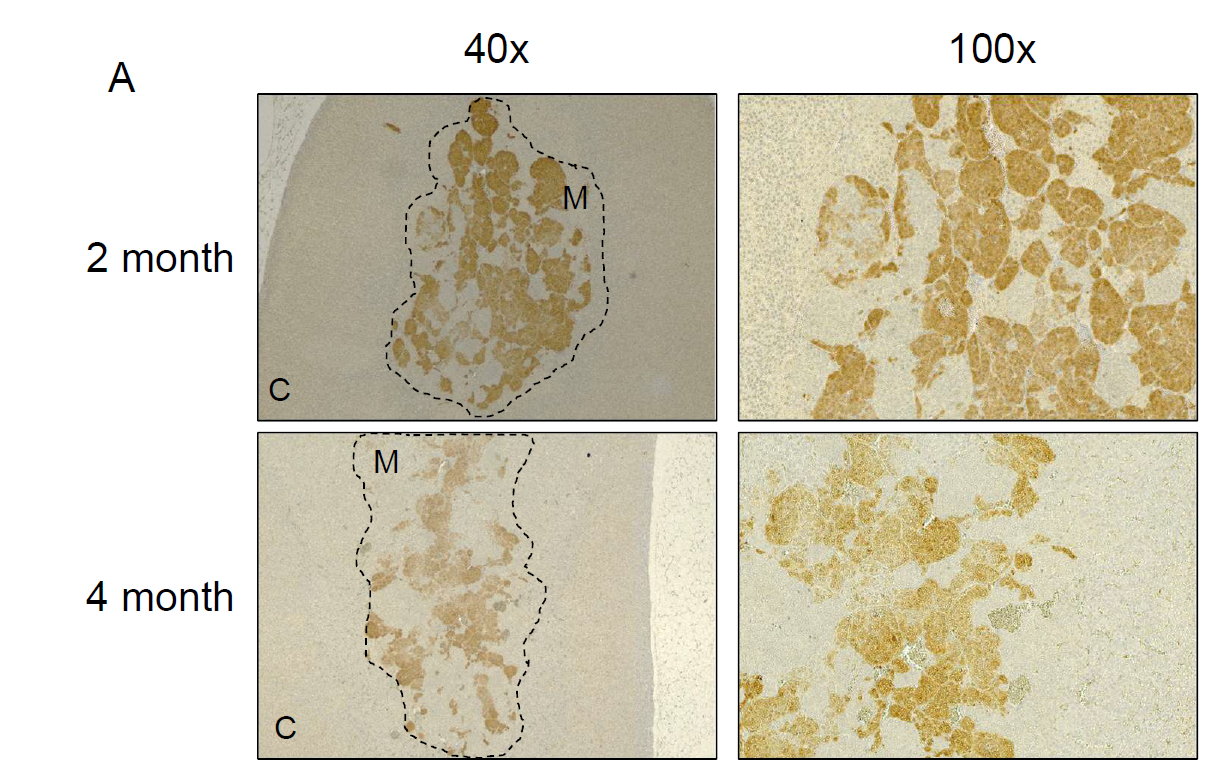


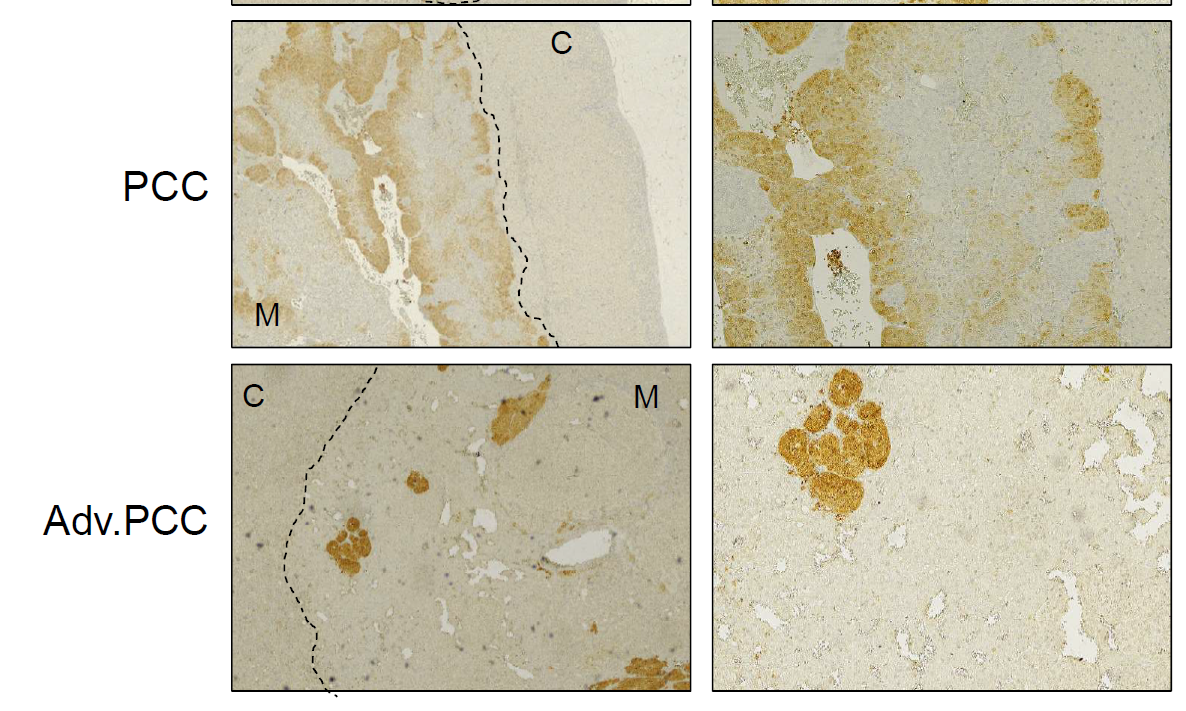


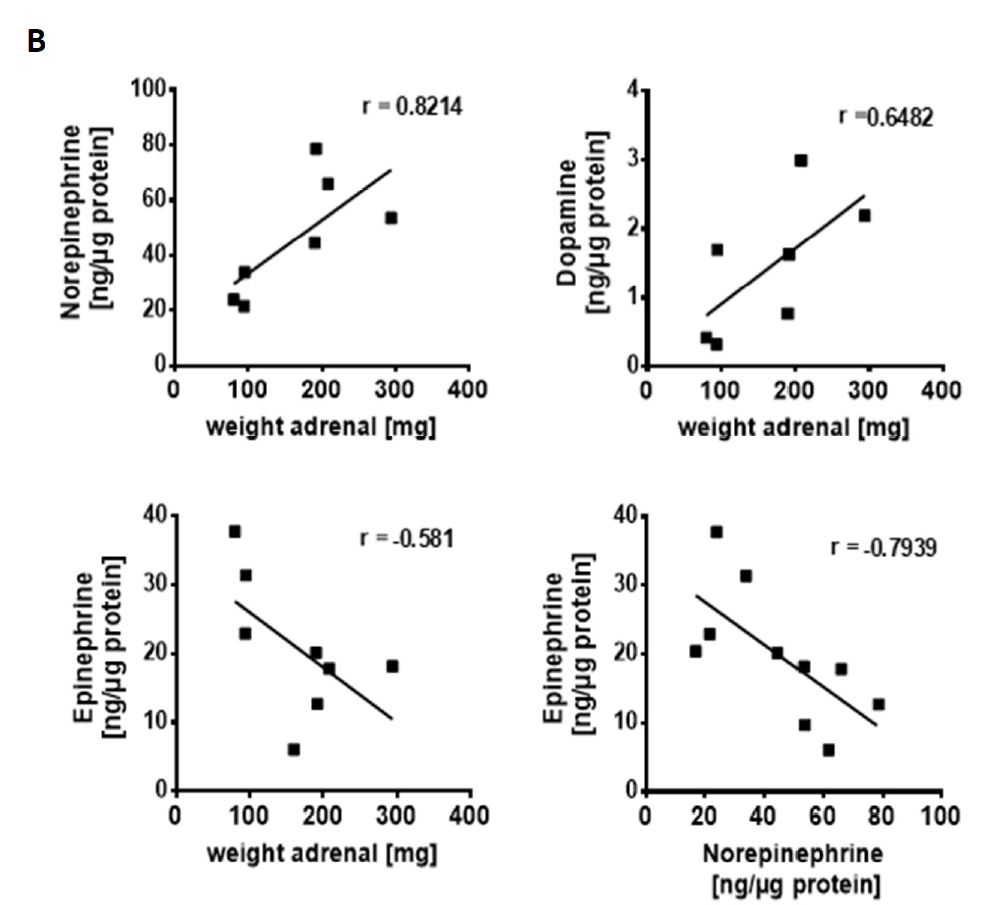


**Figure S1.** PNMT expression and catecholamine profile of MENX PCCs. (**A**)Adrenals from MENX rats at different ages were stained with a PNMT-specific antibody. Representative examples are shown. The proportion of PNMT-positive cells decreased with tumorigenesis. M, medulla; C, cortex. (**B**) Correlation of catecholamine concentration to adrenal weight. NE and DA concentration increased with the weight of the tumor tissue. In contrast EPI drops. The EPI concentration is strongly inverse correlated to the NE concentration in the tumor tissues (depicted is Spearman-Correlation).


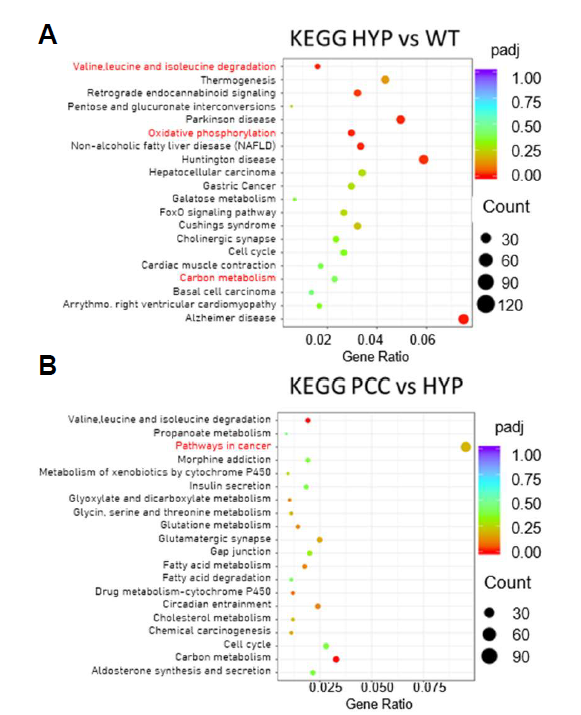


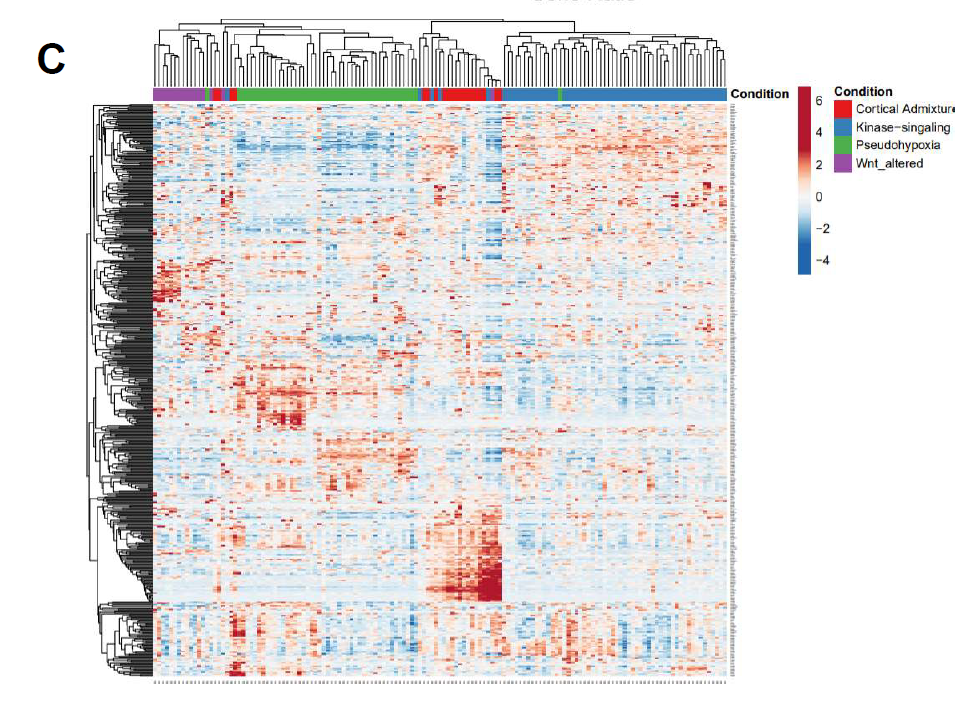


**Figure S2.** Analysis of rat transcriptome and Human TCGA PPGL clustering with the 500 gene list. (**A**–**B**) Wildtype (WT, *n* = 5), Hyperplasia (HYP, *n* = 5), Pheochromocytoma (PCC, *n* = 5) were analysed by RNAseq of macroscopic dissected medulla tissue. Top 20 most significantly enriched KEGG pathways based on the differentially expressed genes between rat datasets A) (HYP vs. PCC) and (**B**) (PCC vs. HYP) are depicted in the dot graph. (**C**) Consensus Clustering of TCGA PPGL and heatmap without rat samples (red = Cortical admixture, blue= kinase-signaling, green = pseudohypoxia, purple = Wnt-activated). The selected subset of 500 genes is sufficient to cluster the human samples into the previously identified cluster with few exceptions mainly by the coritcal admixture cluster. An enlarged figure can be found in a seperate file, Figure S2C.


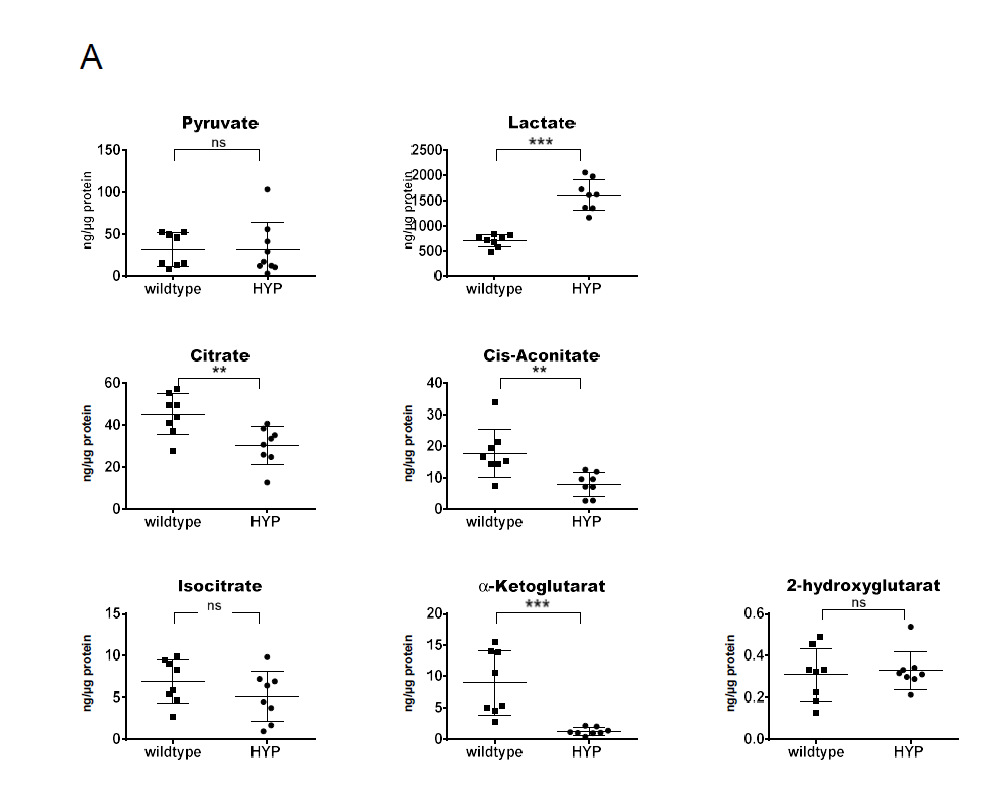


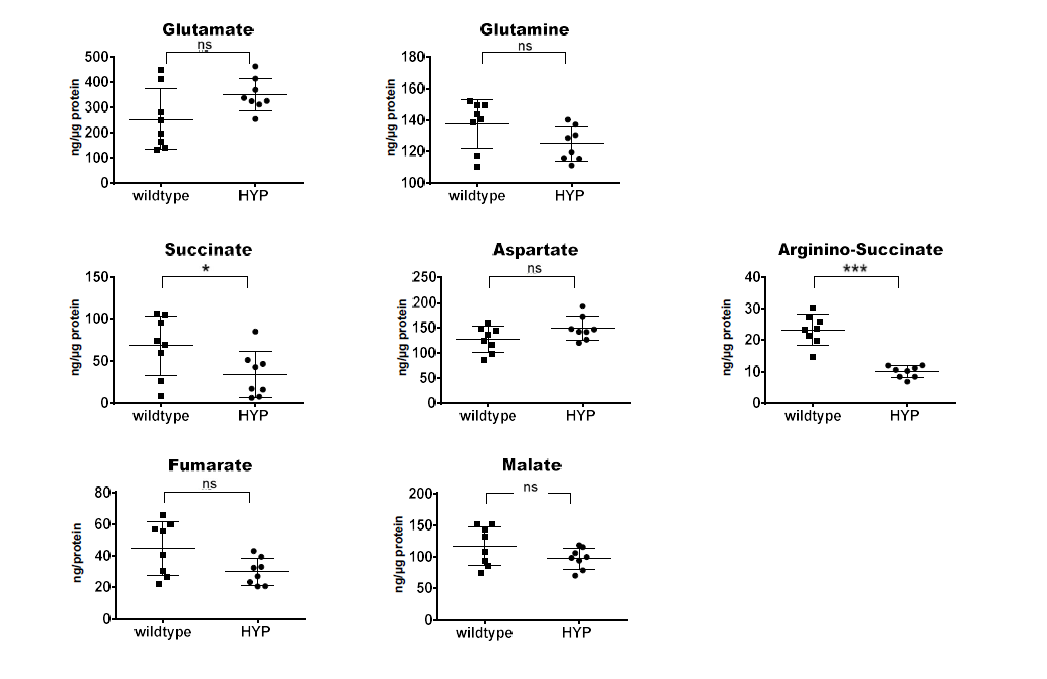


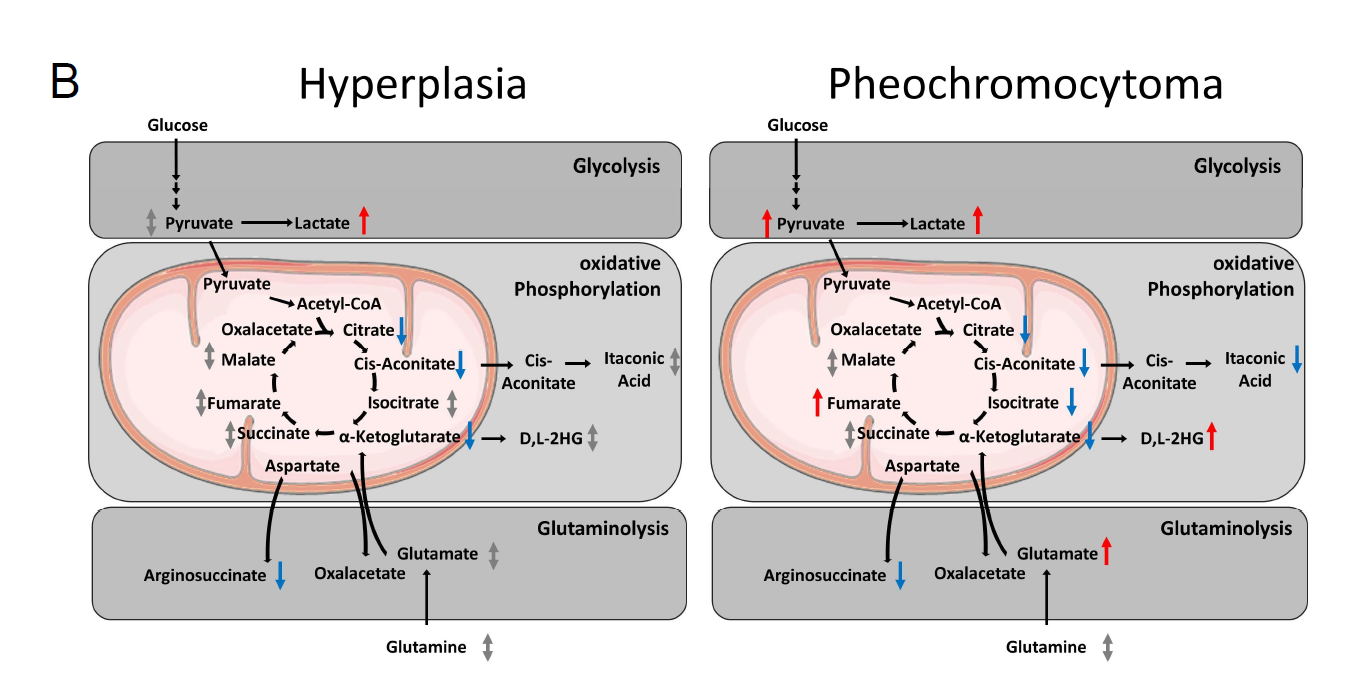


**Figure S3.** Krebs cycle changes in hyperplastic MENX PCCs. (**A**) Metabolomic analysis of TCA-cycle metabolites in adrenal medulla of WT (*n* = 8) and HYP (*n* = 8) at 4–5 months of age was done by LC-MS/MS. Measurements are reported as ng/mg of total protein. Each symbol represents one animal: black square = WT; black dot = HYP. Data are expressed as mean ± SD. *p*-values were calculated by Mann-Whitney-Test. (***, *p* < 0.001; **, *p* < 0.01; *, *p* < 0.05; ns, not significant. Already at the stage of hyperplasia, there is a decrease in several metabolites of the TCA cycle, while Lactate production is increased. (**B**) Scheme illustrating the changes found in TCA-metabolites in MENX rats at 4–5 (HYP) and 7–9 (PCC) months of age. Red arrows indicate increased metabolite levels, blue arrows indicate decreased levels. Many changes seen at HYP became more pronounced during tumor progression.


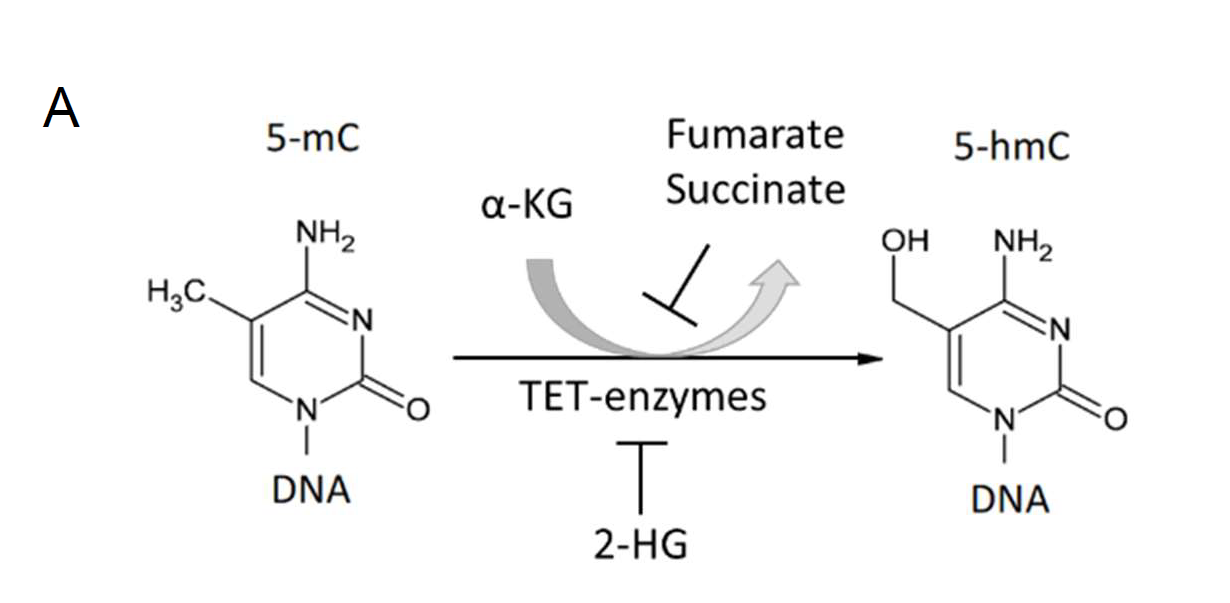


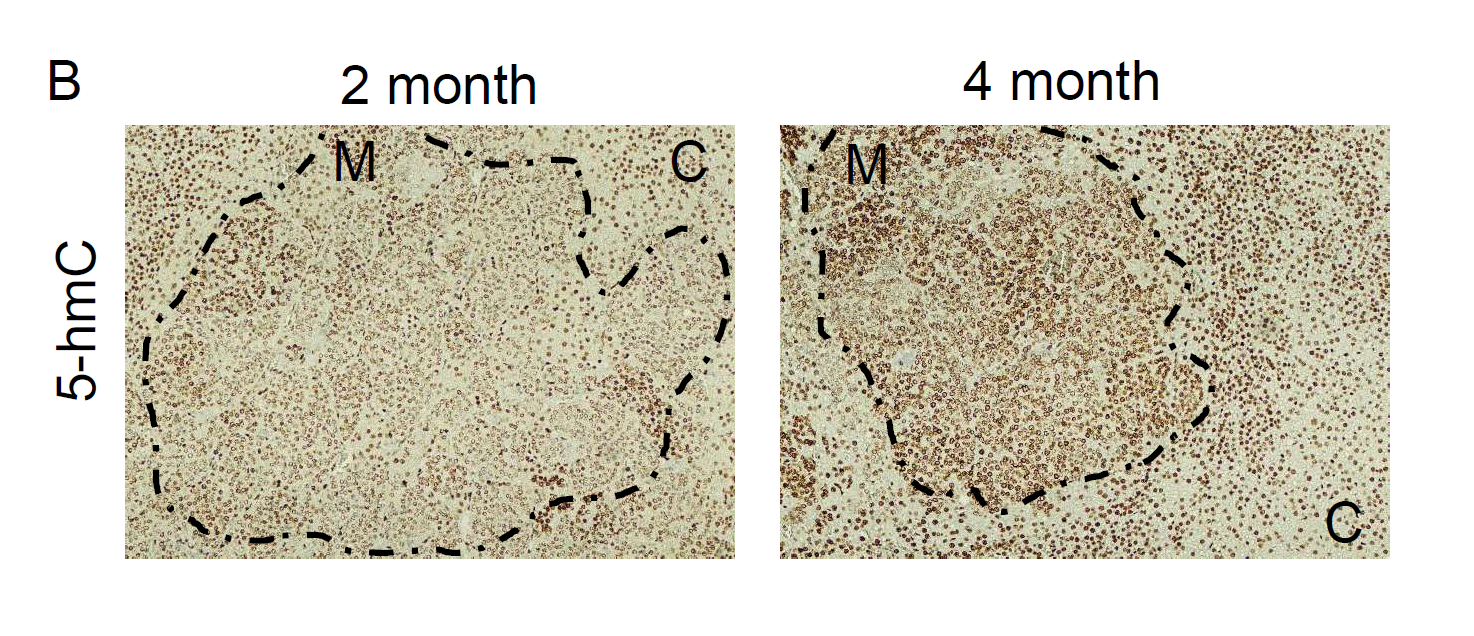


**Figure S4.** TET enzymes activity and 5-hmC expression in MENX rat adrenals. (**A**) Scheme illustrating the enzymes (TET) catalyzing the oxidation of 5-mC to 5-hmC and their inhibitors. (**B**) IHC staining for 5-hmC at 2 month and 4 month of age. M = medulla, C = cortex. 5-hmC staining was positive in adrenal medullary cells in animals before the onset of PCC. Original magnification: 100×.


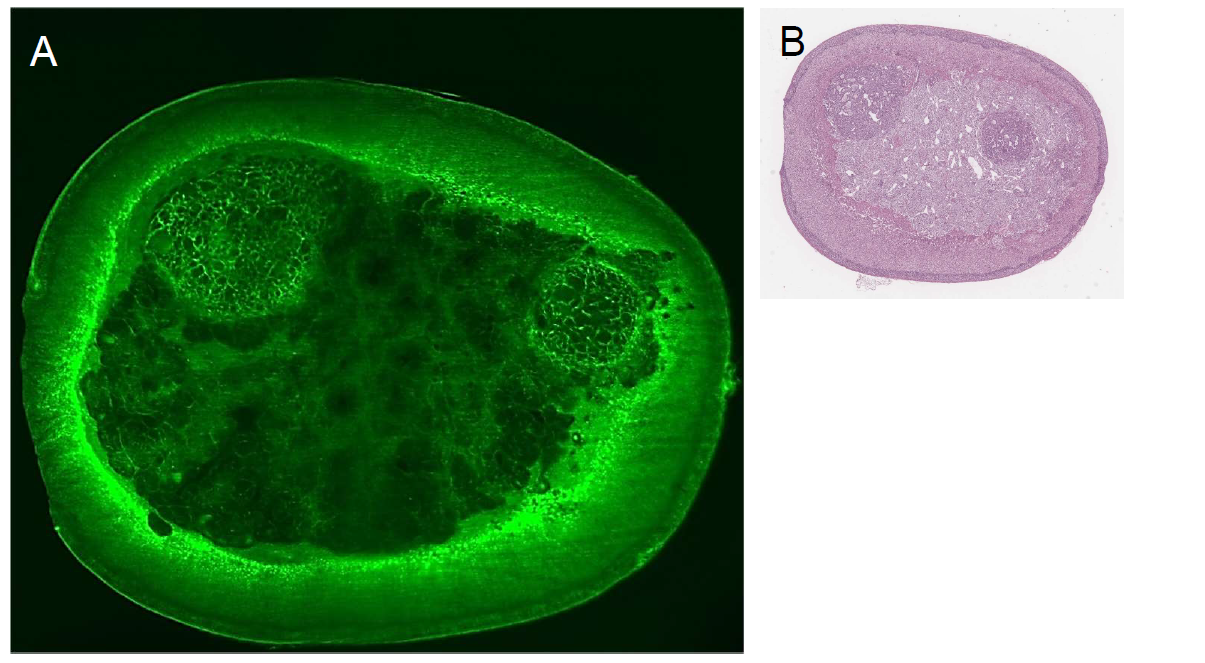


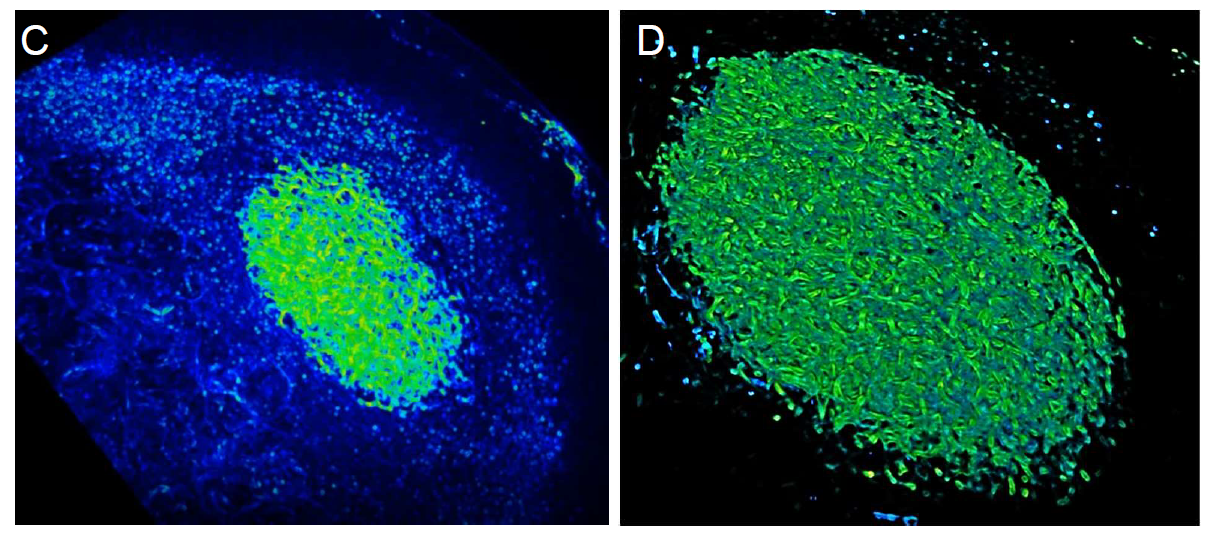


**Figure S5.** Structure of the rat PCC vasculature. Rats were injected with T-Lectin to visualize endothelial cells, and 6 h later sacrificed for organ collection. (**A**) Cross-section of a T-Lectin-stained (green) MENX adrenal gland. Two PCC nodules are visible. (**B**) H&E staining of the gland shown in (**A**). (**C**) PCC nodule stained with T-Lectin (green). Cell nuclei were counterstained with DAPI. This is the nodule shown in the video. (**D**) Higher magnification of the nodule in C. Original magnification: (**A**,**B**) 20×. Panels (**C**,**D**) derive from the video.


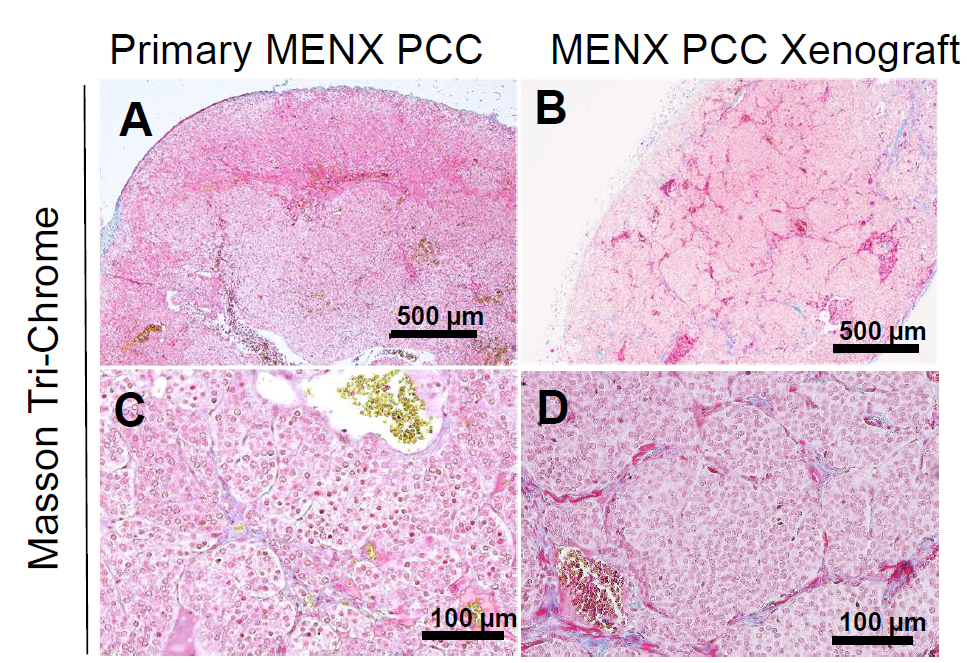


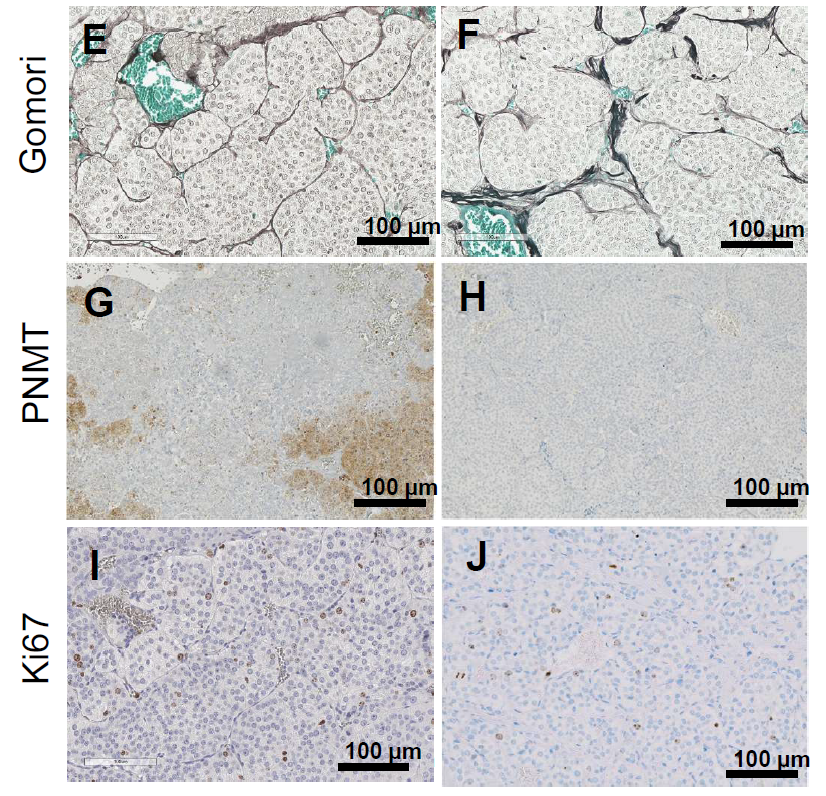


**Figure S6.** Comparison of the morphology of primary MENX PCCs and tumors derived from xenografted MENX PCC cells. (**A**–**D**) Masson-Tri-Chrome staining, depicting erythrocytes in yellow and collagen in blue. (**E**–**F**) Gomori-Silver Methanamin stains Reticulin in black and erythrocytes in green. (**G**–**H**) PNMT staining shows the lack of PNMT in the xenografted tissue, while there are some residual cells positive in the primary tumor. (**I**–**J**) Ki67 staining indicating proliferating cells. Although there were some variations between individual tumors of the primary and xenografs, the overall charcteristics and morphology was maintained after injecting the primary tumor cells subcutaneously into mice.
